# Classification, function, and advances in tsRNA in non-neoplastic diseases

**DOI:** 10.1038/s41419-023-06250-9

**Published:** 2023-11-16

**Authors:** Liou Zhang, Jie Liu, Yang Hou

**Affiliations:** 1 Department of Radiology, Shengjing Hospital of China Medical University, Shenyang, China; 2 Translational Research Experiment Department, Science Experiment Center, China Medical University, Shenyang, China; 3 Department of Radiology, Shengjing Hospital of China Medical University, Shenyang, 110004 China

**Keywords:** Non-coding RNAs, Diseases, Biomarkers, Cell biology

## Abstract

tRNA-derived small RNAs (tsRNAs) are non-coding small RNAs produced by specific endonucleases following the processing and splicing of precursor or mature tRNAs upon starvation, oxidative stress, hypoxia, and other adverse conditions. tRNAs are classified into two major categories, tRNA fragments (tRFs) and tRNA-derived stress-induced small RNAs (tiRNAs), based on differences in splice sites. With the development of high-throughput sequencing technologies in recent years, tsRNAs have been found to have important biological functions, including inhibition of apoptosis, epigenetic regulation, cell–cell communication, translation, and regulation of gene expression. Additionally, these molecules have been found to be aberrantly expressed in various diseases and to be involved in several pathological processes. In this article, the classification and nomenclature, biological functions, and potential use of tsRNAs as diagnostic biomarkers and therapeutic targets in non-neoplastic diseases are reviewed. Although tsRNA research is at its infancy, their potential in the treatment of non-tumor diseases warrants further investigation.

## Facts

- tRNA-derived small RNAs (tsRNAs) are a type of small non-coding RNA originating from different regions of tRNA, including the 5′ end, 3′ end, and internal regions.
- tsRNAs have diverse functions in various biological processes. They can regulate gene expression, post-transcriptional modifications, cell proliferation, and apoptosis. Additionally, tsRNAs are involved in stress responses, DNA damage repair, and immune responses.
- tsRNAs show great potential as biomarkers for diagnosing and monitoring diseases. They have been detected in various body fluids, such as the blood, urine, and saliva, and their expression patterns are altered in different diseases, including cancer, neurodegenerative disorders, and cardiovascular diseases.

## Open questions

- What are the specific mechanisms by which tsRNAs regulate gene expression and other cellular processes?
- How do tsRNA expression patterns vary across tissues and cell types?
- While some correlations between tsRNA and diseases have been identified, the exact roles and mechanisms of tsRNA in diseases remain unclear. Can tsRNAs be therapeutically targeted for the treatment of diseases?

## Introduction

Most tRNAs comprise 70–90 nucleotides (nt) folded in an “inverted L”-shaped three-dimensional structure. Their central role is to transfer specific amino acids to ribosomes, during translation, for mRNA-guided protein synthesis [1]. In addition, precursor or mature tRNAs can be spliced to form various small RNAs, collectively referred to as tRNA-derived small RNAs (tsRNAs). tsRNA, short hairpin RNA (shRNA), and small interfering RNA (siRNA) are small RNA molecules involved in gene regulation; however, they differ in their origin, processing, and mechanism of action. The tsRNAs are derived from tRNAs, while shRNAs and siRNAs are artificially synthesized or naturally generated molecules used in RNA interference experiments[2–4]. Nucleotides produced from tRNA degradation were first detected in 1977 in the urine of cancer patients, using -aminoisobutyric acid as a probe [5]. However, at the time, they were believed to be products of random tRNA degradation and attracted limited attention. In 2009, Fu et al. [6] obtained tRNA fragments generated by tRNA cleavage in the anticodon loop, which were present in many cell lines and different mouse tissues. Angiogenin (ANG) was identified as the endonuclease that cleaves tRNA. Recently, an increasing body of research has confirmed the widespread and stable expression of tsRNAs, which are conserved across species. In addition, tsRNA is associated with the cellular stress response [7], indicating that tsRNAs enable cells to respond rapidly to external stimuli and are involved in cellular adaptation and regulation, thus maintaining cellular homeostasis and survival. Moreover, tsRNAs can regulate gene expression, including that of major biological processes such as cell proliferation, differentiation, and apoptosis. Numerous studies have identified abnormally-expressed tsRNAs in patients with various malignant cancers [8–12], suggesting that tsRNAs can be used both as diagnostic markers for diseases and as therapeutic targets. However, as a novel, non-coding, functional, small RNA, its research value in non-neoplastic diseases should not be neglected. In summary, tsRNA is a class of small RNAs produced from tRNA molecules that play an important role in cells. Focusing on the importance of tsRNAs deepens our understanding of the basic biological processes of cells, elucidates the mechanisms of diseases, and provides new directions and avenues for their diagnosis and treatment.

## Classification and naming conventions of tsRNA

**Fig. 1:**
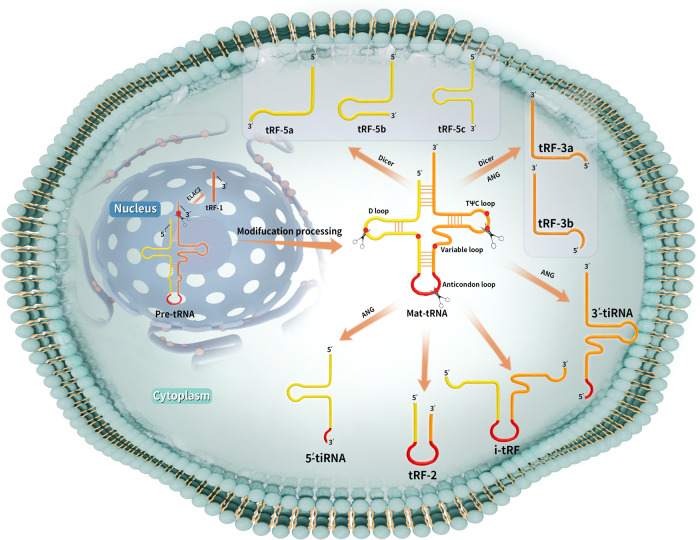
The tsRNAs are classified as tRFs or tiRNAs depending on their cleavage site. tRF-1 is derived from the 3′ end of the precursor tRNA and is produced following cleavage by ELAC2. tRF-3 is derived from the 3′ end of mature tRNA and is produced by cleavage of the T-loop by Dicer or ANG. tRF-3 is further classified into two subtypes based on the cleavage position. tRF-5 is derived from the 5′ end of mature tRNA and results from Dicer-medicated cleavage and can be further classified into three subtypes based on length. tRF-2 is derived from cleavage of mature tRNA within the anticodon loop. i-tRF is derived primarily from the internal region of mature tRNA (between the D- and T-loops) and includes the anticodon loop. tiRNAs are generated from ANG-mediated cytoplasmic cleavage of mature tRNAs into 5′ tiRNAs and 3′ tiRNAs. ANG Angiogenin, ELAC2 Zinc phosphodiesterase ELAC protein 2, Mat-tRNA Mature tRNA, Pre-tRNA Precursor tRNA, tRF tRNA fragments, tiRNA tRNA-derived stress-induced small RNA.

tsRNAs are classified into two major categories, tRNA fragments (tRFs) and tRNA-derived stress-induced small RNAs (tiRNAs), based on the cleavage site (Fig. 1). tRFs are fragments composed of 14–30 that originate from the ends of precursor or mature tRNAs. The two-dimensional structure of the three-leaf clover-shaped tRNA comprises three hairpin loops: a D loop, anticodon loop, and TψC loop [13, 14]. These can be classified into five categories based on the cleavage site: tRF-1, tRF-3, tRF-5, tRF-2, and i-tRF [15–17].

tRF-1 is derived from the 3′ end of the precursor tRNA and is produced following cleavage by ELAC2. tRF-1 is also known as 3′ U-tRF because it contains the poly-U RNA polymerase III transcription termination sequence [18, 19]. tRF-3 is derived from the 3′ end of mature tRNA and is produced by cleavage of the T-loop by Dicer or ANG. tRF-3 is further classified as tRF-3a or tRF-3b depending on its length [20]. Cleavage between nucleotides 58 and 59 results in tRF-3a, with a length of 18 nt; cleavage between nucleotides 54 and 55 results in tRF-3b, with a length of 22 nt [21]. tRF-5 is derived from the 5′ end of mature tRNA and results from Dicer-mediated cleavage upstream of the anticodon loop. tRF-5 has three isoforms. tRF-5a (14–16 nt), tRF-5b (22–24 nt), and tRF-5c (28–30 nt) [22]; The tRF-5 species identified to date and present in the tRF database (tRFdb) are larger than tRF-3 and tRF-1.

tRF-2 is derived from cleavage of mature tRNA within the anticodon loop [23]. i-tRF is derived primarily from the internal region of mature tRNA (between the D and TψC loops). Thus, i-tRF includes the anticodon loop and is produced following cleavage by an unknown endonuclease [24]. These tRNA fragments can be further subclassified into three types, based on the location of the 5′ end: D-tRF, A-tRF, and V-tRF [25]. tRF-2 and i-tRF production is thought to be associated with hypoxic stress [26]. Some studies combine these tsRNAs into a single class; however, few reports have focused on both tRF-2 and i-tRF, and it is currently believed that their production may be associated with hypoxic stress[26]. i-tRF is longer than tRF-2, and cleavage sites for both are located in the D and TψC loops, but neither extends to the 5′ and 3′ ends. It has been suggested that the length of these tsRNAs is closely associated to specific disease states [27]; however, the exact mechanisms remain unclear.

tiRNAs are slightly longer than tRFs, typically 31–41 nt in length; they are also known as “tRNA halves.” When cells are subjected to stress conditions, such as hypoxia or nutrient deficits, the anticodon loop of mature tRNA is cleaved by ANG into 3′ and 5′ tiRNAs [28, 29]. The 3′ tiRNA ends at the 3′ end of the mature tRNA, while 5′ tiRNA ends at the 5′ end of the mature tRNA. These tiRNAs have different sequence lengths, with 3′ tiRNAs containing 36–41 nt and 5′ tiRNAs 31–36 nt.

tsRNAs have been classified into three categories, based on the splice site [30]. The first category is 5′ tsRNA, which starts at the 5′ end and terminates at various sites between the D and anticodon loops of the mature tRNA; these include tRF-5s and 5′ tiRNAs from the original classification [31]. The second category is 3′ tsRNA, which is derived from the 3′ end of mature tRNA, containing CCA tail sequences. The 3′ tsRNAs start at the 3′ end and terminate at different sites between the TψC and anticodon loops of mature tRNAs and include tRF-3s from the original classification [31]. The third category includes tRF-1 and i-tRFs [28].

In 2015, the tRFdb [32] was established, the first database for tRFs, which includes tsRNAs from several species, namely human and mouse. The tRFs present in the database can be identified by sequence number or ID, which can be used to retrieve the tRF name and supporting experimental data. In recent years, other tsRNA databases have been developed, including tDRmapper, MINTbase, and tsRBase, providing relevant information to support tsRNA research, which are convenient resources to support tsRNA research. However, a uniform naming convention for tsRNAs has not been established. tsRNA nomenclature should include the following [33]: (1) specific classification of the tsRNA, such as tRF-1, tRF-3, 3′ tiRNA, or 5′ tiRNA; (2) chromosome number; (3) tRNA number; (4) corresponding amino acid; and (5) anticodon carried by the tRNA. For example, the sequence tRF-1001 in tRFdb corresponds to a tRF-1, chromosome 10, tRNA number 2, and corresponding amino acid and its anticodon are Ser-TGA.

## tsRNA function

**Fig. 2:**
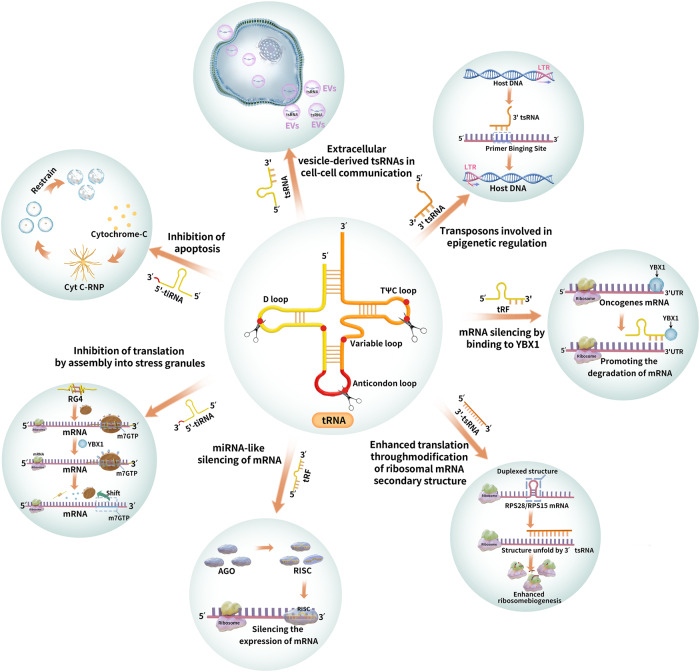
The tsRNAs derived from tRNA cleavage are involved in a variety of biological functions, including mRNA silencing, inhibition of apoptosis, regulation of protein production at the translational level, regulation of epigenetics, and cell–cell communication via exosome-derived tsRNAs. AGO Argonaute, RISC RNA-induced silencing complex, UTR Untranslated region, RG4 RNA G-quadruplex, YBX1 Y-box binding protein 1, EVs Extracellular vesicles, Cyt C Cytochrome C, RNP Ribonuclear protein, tRF tRNA fragments, tiRNA tRNA-derived stress-induced small RNA.

Although the specific mechanisms involved in the biological functions of tsRNAs remain unclear, multiple studies have confirmed that they play important roles in mRNA silencing, translation regulation, apoptosis inhibition, cell–cell communication, and epigenetic regulation (Fig. 2) [33–58].

### miRNA-like silencing of mRNA

**Fig. 3:**
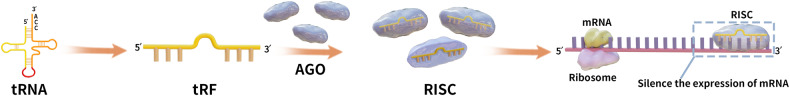
Similar to miRNAs, tRFs can inhibit the expression of target genes by binding to the 3′ UTR sequence of the target gene mRNA via Argonaute (AGO) proteins to form the RNA-induced silencing complex (RISC). AGO Argonaute, RISC RNA-induced silencing complex, UTR Untranslated region.

miRNAs interact with target mRNAs through base pairing and participate in gene regulation by inhibiting target gene expression. miRNA-like silencing of tsRNA involves the use of small RNA molecules derived from tRNA fragments to regulate gene expression. Whereas clustered regularly interspaced short palindromic repeats(CRISPR) is a gene-editing tool that allows for precise modifications of the DNA sequence. Both mechanisms have different functions, involvement, and specificity in regulating gene expression. Unlike CRISPR gene editing, which makes precise alterations in DNA, tsRNAs achieve gene silencing endogenously with an miRNA-like mechanism of action through complementary binding to the 3ʹ untranslated region (UTR) of the target mRNA molecule [34–36]. tRFs possess seed sequences similar to those of miRNAs and act like miRNAs by binding to the argonaute (AGO) protein to form an RNA-induced silencing complex (RISC). These sequences are complementary to the 3′ untranslated region (UTR) sequence of the target gene mRNA, which results in the inhibition of target mRNA expression (Fig. 3) [37–39]. tsRNA binds to AGO proteins from a wide range of species, including ciliates, plants, silkworms, flies, mice, the common marmoset, and humans [40]. tRF-1 typically interacts with AGO3 and AGO4, and tRF-5 and tRF-3 interact with AGO1, AGO3, and AGO4 [33]. Tong et al. [41] found that tRF-3017A inhibited the expression of the downstream gene NELL2 in patients with gastric cancer by forming an RISC with AGO, thereby suppressing tumor migration and invasion.

### Inhibition of translation by assembly into stress granules

**Fig. 4:**
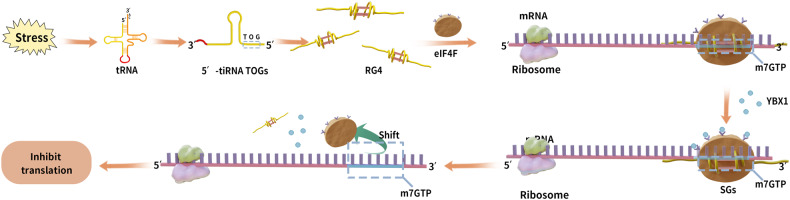
The end of the 5′ tiRNA contains a 5′ TOG motif that can be assembled into an RNA G-quadruplex (RG4) structure. This, in turn, acts as a translational repressor by interacting with the YBX1 protein, inducing the formation of stress granules (SGs), which leads to the translocation of eIF4F from the m7GTP cap of the mRNA. eIF4F Eukaryotic initiation factor 4F, RG4 RNA G-quadruplex, SG Stress granule, YBX1 Y-box binding protein 1.

In eukaryotic cells, phosphorylation of the eukaryotic initiation factor (eIF)2 promotes the formation of stress granules (SGs) that delay translation, a stress-response mechanism against environmental stress [42]. The 5′ tiRNA can inhibit translation by synthesizing and promoting the assembly of SGs in an eIF2 phosphorylation-independent manner. The 5′ tiRNA-Ala contains a 5′ TOG motif at its end that can assemble into an RNA G-quadruplex (RG4) that binds eIF4F. This promotes the release of eIF4F from the m7GTP cap of the mRNA, leading to translation repression. The RG4, formed from 5′ tiRNA assembly, can interact with the Y-box binding protein 1 (YBX1) to induce SG formation. The tiRNA-mediated SG formation is dependent on YBX1 activity and independent of eIF2 phosphorylation (Fig. 4) [43, 44].

### mRNA silencing by binding to YBX1

tsRNA regulates gene expression by binding to the YBX1 protein, independent of SG formation. YBX1 binds to the 3′ UTR of oncogene mRNA, resulting in increased transcriptional stability and enhanced expression of oncogenic proteins. The binding of tRFs to YBX1 leads to competitive inhibition of oncogene expression and stability. This post-transcriptional silencing is sequence specific, because these tRF fragments share a common motif corresponding to the YBX1 recognition sequence, known as the CU-box motif. A class of tRFs derived from tRNA-Glu, tRNA-Asp, tRNA-Gly, and tRNA-Tyr has been shown to inhibit gene expression by competing with oncogene mRNA to bind the YBX1 protein (an RNA-binding protein), thereby suppressing the progression of breast cancer [45]. Additionally, tRFs bind YBX1 in a sequence-specific manner to inhibit gene expression, a mechanism different from that involved in SG assembly to inhibit translation.

### Enhanced translation through modification of ribosomal mRNA secondary structure

**Fig. 5:**
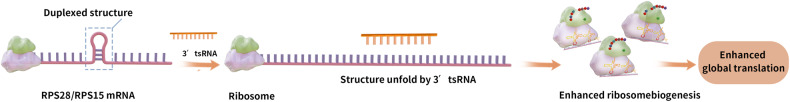
3′ tsRNA enhances translation by binding two ribosomal protein mRNAs (RPS28 and RPS15) and unfolding their secondary structures, leading to increased ribosome production.

Binding of 3′ tsRNA to ribosomal mRNA can cause its secondary structure to unfold and enhance translation. Kim et al. [46] found that a LeuC-AG 3′ tsRNA, which is approximately 22 nt in length, significantly increased cell viability. Inhibition of this Leu-CAG 3′ tsRNA resulted in a reduction in the number of ribosomes and induction of apoptosis. In addition, LeuCAG 3′ tsRNA binds the mRNA of two ribosomal proteins (RPS28 and RPS15) via base pairing, leading to the unfolding of the mRNA secondary structure, promoting translation and the formation of 40 S ribosomes (Fig. 5). Leu-CAG 3′ tsRNA does not affect the translation of mRNAs of other ribosomal proteins (e.g., RPS9/RPS14), suggesting that 3′ tsRNA-mediated changes in mRNA secondary structure are essential for translation regulation.

### Transposons involved in epigenetic regulation

Transposable elements, known as genomic parasites, are genomic DNA fragments that can move autonomously or non-autonomously [47]. Transposons are mobile genetic elements that can cause genetic changes and affect nearby genes, whereas other effectors involved in epigenetic regulation primarily act by modifying DNA or histones to regulate gene expression. They have different mechanisms, biological roles, and modes of inheritance in regulating gene expression and genome stability [48, 49]. The most active transposons in mice are long terminal repeats-retrotransposons, also known as endogenous retroviruses (ERVs). Schorn et al. [50] identified abundant tRFs in mouse stem cell lines. A 3′ tsRNA, 18 nt in length, competes with mature tRNA to bind the primer-binding site of ERVs, thereby blocking its reverse transcription and transposon mobility. This, in turn, affects ERV cDNA synthesis. This ability to inhibit transposons is highly conserved, suggesting that tsRNA could be a novel epigenetic regulator [50].

### Extracellular vesicle-derived tsRNAs in cell–cell communication

Extracellular vesicles (EVs) are encased in a lipid bilayer and released from cells. EVs are classified into three main categories based on their size and origin: exosomes, microvesicles, and apoptotic bodies [51, 52]. tsRNAs are highly expressed in cellular and plasma exosomes [53, 54] and EV-derived tsRNAs are involved in mediating cell–cell communication. For example, high levels of 5′ tRFs, exceeding those of other small RNAs, have been found in activated T cell-derived EVs, and their inhibition enhances T cell activation [55]. This T cell activation is achieved through the formation and secretion of multivesicular bodies, which results in subsequent downregulation of 5′ tRFs in EVs. This suggests that tRFs present in secreted EVs are involved in T cell activation through a signaling-regulated mechanism [55].

### Inhibition of apoptosis

**Fig. 6:**
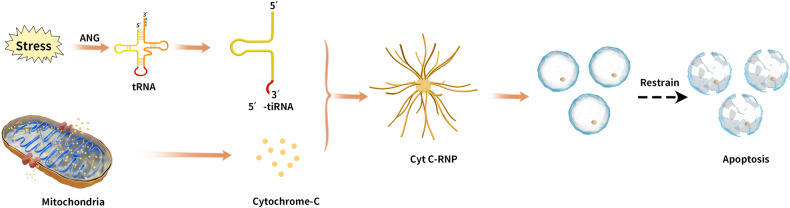
Under stress conditions, tiRNA formed from ANG cleavage can inhibit apoptosis by binding to cytochrome C released from mitochondria to form the Cyt C-RNP complex. ANG Angiogenin, Cyt C Cytochrome C, RNP Ribonuclear protein.

Previous studies have shown that mature tRNA is involved in translation and apoptosis inhibition via binding to cytochrome C (Cyt C), which blocks the formation of apoptotic bodies and activation of caspase-9 [56]. In contrast, tiRNAs formed from ANG cleavage of tRNA under stress conditions inhibit apoptosis by binding to Cyt C released from mitochondria to form the Cyt C-ribonuclear protein (RNP) complex (Fig. 6) [57]. Tao et al. [58] found that tRNA expression was upregulated by 5′ tiRNA-His-GTG in colon cancer tissue and that inhibition of 5′ tiRNA-His-GTG expression induced apoptosis. Additionally, they showed that 5′ tiRNA-His-GTG is regulated through the hypoxia-inducible factor/ANG axis. A major target of 5′ tiRNA-His-GTG is large tumor suppressor kinase 2, which enables 5′ tiRNA-His-GTG to inactivate the Hippo signaling pathway, promoting the expression of genes with pro-proliferation and anti-apoptotic activities [58].

## Research progress in tsRNA and non-neoplastic diseases

### tsRNAs and neurological disorders

tsRNAs are expressed in different regions of the brain at varying levels. In primates, tsRNA expression is approximately six-fold higher in the hippocampus than in other brain regions and other organs, with the most abundant types being 5′ tiRNA-Gly-GCC and 5′ tiRNA-Glu-CTC [59]. Growing evidence demonstrates that specific tsRNAs play important roles in the pathogenesis of developmental, degenerative, and behavioral neurological disorders [59–72]. Expression of some tsRNAs is significantly altered in the hippocampus of patients with Alzheimer’s disease (AD), which is the most common type of dementia, characterized by prominent pathological manifestations and biochemical alterations in the hippocampus [60]. One of the hallmarks of AD is the increased expression of -amyloid that forms neurotoxic plaques between neurons in the brain, the underlying cause of AD [61]. In patients with AD, the most highly expressed tsRNA is tRF-5, which has a length of 30–40 nt [62, 63]. This tRF (named AS-tDR-013428) is involved in the development of AD by targeting Rpsa, a gene implicated in the production and internalization of neurotoxic -amyloid through an miRNA-like action [64]. These findings suggest that tRF-5 is a novel signal in the pathogenesis of AD (Table 1).

**Table 1:** List of non-neoplastic diseases associated tsRNAs.

Disease	Name	Type	Expression	Mechanism/Function	References
AD	30–40nt tRFs	trf-5	UP	Progression biomarker	[62, 63]
AD	AS-tDR-013428	trf-5	UP	Diagnostic biomarker	[64]
PD	AS-tDR-011775	trf-5	UP	Diagnostic biomarker	[64]
ALS	tiRNA(Ala)、tiRNA(Cys)	5′-tiRNA	UP	Inhibit translation	[68]
ALS	5′ValCAC	5′-tiRNA	UP	Prognostic biomarker	[70]
Epilepsy	5′GlyGCC, 5′AlaTGC、5′GluCTC	trf-5	UP	Predict seizure risk	[72]
Fertility	tRNAGln-TTG	trf-2	UP	Diagnostic biomarker	[80, 81]
AS	tRF-Gly-GCC-009、tRF-Gly-GCC-008	tRF-5c	UP	Biomarker and treatment target	[82]
	tRF-Pro-AGG-006、tRF-Pro-AGG-005	tRF-5c	Down	Biomarker and treatment target	
Myocardial ischemia	5′ tRNA-Val-AAC	5′-tiRNA	UP	Unknown	[6]
Myocardial hypertrophy	tRNA-Gly-CCC	trf-1	UP	Inhibite mRNA expression	[90]
Fracture healing	Lys-CTT-5′end、Lys-TTT-5′end	5′tiRNA	Down	Biomarker for tissue regeneration	[91]
	Lys-TTT、Lys-CTT	i-tRF	Down		
	His-GTG-5′	5′tiRNA	UP		
	His-GTG	i-tRF	UP		
Osteoporosis	tRF-25、tRF-38、tRF-18	trf-3	UP	Diagnostic biomarker	[93]
Osteoarthritis	tRF-5009A	trf-5	Down	Inhibite mRNA expression	[95]
SLE	tRF-His-GTG-1	trf-5	UP	Diagnostic biomarker	[98]
IgAN	tRF-Val-AAC-007	trf-5	UP	Biomarker and treatment target	[105]
	tRF-Gln-CTG-010、tRF-Thr-AGT-007	trf-3	UP		
	tRF-Tyr-GTA-011、tRF-Ala-AGC-063				
	tiRNA-Val-TAC-004	5′-tiRNA	Down		
	tRF-Gly-CCC-005、tRF-His-GTG-006	trf-5	Down		
Obesity	tsRNA-16902	trf-3	UP	Regulate adipogenic differentiation	[107]
Obesity	tRNA-Glu-TTC	5′-tiRNA	UP	Inhibite mRNA expression	[108]
HBC/HCV	5′tsRNAGly、5′tsRNAVal	5′-tiRNA	UP	Unknown	[109]
ALI	tRF-22-8BWS7K092	trf-3	UP	Induce ferroptosis	[113]
RILI	tRF-Gly-GCC	trf-5	UP	Inhibite cell proliferation and promote apoptosis	[116]

Parkinson’s disease (PD), another neurodegenerative disease, is associated with degeneration of dopaminergic neurons and -synuclein in the substantia nigra [65]. High levels of tRF has been found from the prefrontal cortex, cerebrospinal fluid, and serum of patients with PD with high sensitivity (89–100%) and specificity (79–98%), relative to case controls. This suggests that tRF is a potential novel non-invasive diagnostic biomarker for PD [66]. In the mouse brain [64], tRF-5 (named AS-tDR-011775) targets the Mobp gene and is involved in the pathogenesis of PD, affecting axon morphology.

Amyotrophic lateral sclerosis (ALS) is characterized by muscle weakness due to the degeneration of motor neurons [67]. As discussed, upon cellular stress, 5′ tiRNA-Ala and 5′ tiRNA-Cys are produced through cleavage of tRNA by ANG. These species have been found to facilitate SG formation by assembling into an RG4 structure that permits spontaneous entry of SGs into motor neurons, triggering a neuroprotective response [68]. Mutations in the ANG gene are associated with ALS [69], suggesting that tiRNA can mediate its pathogenesis. Using a mouse model of ALS, Hogg et al. [70] found that 5′ t-RNA-Val-CAC was significantly expressed in the mouse serum. In accordance with the experimental model, in clinical serum samples, 5′ tRNA-Val-CAC expression was significantly elevated in patients with slow disease progression, suggesting that this tRNA has potential as a prognostic biomarker for ALS.

Epilepsy is a common and serious neurological disorder, affecting nearly 50 million people worldwide. Epilepsy is defined as the transient onset of symptoms due to excessive neuronal activity in the brain [71]. The prediction of epileptic seizures is largely dependent on EEG recordings; therefore, there is clinical interest in the identification of biomarkers that can be used to predict seizures. Hogg et al. [72] sequenced small RNAs in plasma samples collected during video EEG monitoring from patients with focal epilepsy and found that the expression levels of three tRNA fragments (5′ Gly-GCC, 5′ Ala-TGC, and 5′ Glu-CTC) before epileptic seizures were significantly higher than those after epileptic seizures, suggesting that they may serve as novel markers for predicting seizure risk.

### tsRNAs and reproductive system disorders

A study found that injection of sperm heads from high-fat diet-induced obese mice into normal zygotes resulted in F1 mice that developed phenotypes similar to those of the parental mice, such as obesity, glucose intolerance, and insulin resistance, even when given a normal diet. Further analysis revealed substantial changes in the tsRNA expression and RNA modification profiles in the sperm of the obese, paternal mice. This suggests that tsRNAs serve as a carrier of epigenetic information for intergenerational transmission of high fat-induced metabolic disorders [73]. Several studies have confirmed that tsRNAs are abundant in gametes, embryos, epididymis, and seminal vesicles [74, 75]. They participate in gamete maturation, regulate the function of fertilized eggs, influence the embryonic development process, and transmit epigenetic information to offspring, leading to corresponding diseases [76, 77].

Overall, 56% of short-stranded non-coding RNAs in the human sperm are derived from tsRNAs, and they are significantly associated with sperm quality [78]. Notably, tsRNA expression in germ cells is dynamic during spermatogenesis and maturation [79]. Using animal models, Chen et al. [80] demonstrated that seminal plasma-derived exosomes can carry tsRNAs for delivery to spermatozoa. Additionally, tRNA fragments (Gln-TTG) are involved in the early cleavage of preimplantation embryos through the regulation of cell cycle-related gene expression. tRNA-Gln-TTGs have also been shown to be highly associated with human sperm quality, and their overexpression was found to accelerate human embryo development. These findings suggest that human sperm-derived tRNA-Gln-TTG could be used as a potential diagnostic biomarker and clinical therapeutic target for male infertility [81].

### tsRNAs and cardiovascular system disorders

Atherosclerosis (AS) is a chronic inflammatory disease characterized by lipid deposition and accumulation within the intima of the arterial wall followed by endothelial injury, smooth muscle cell proliferation, plaque formation, and stenosis and sclerosis of the vessel wall. One study reported the overexpression of tRF-5cs, tRF-Gly-GCC-009, and tRF-Gly-GCC-008 and significant downregulation of tRF-Pro-AGG-006 and tRF-Pro-AGG-005 in AS tissue samples, compared with the relative expression in controls [82]. In another study using AS tissue samples [83], increased tRF-5a and decreased tRF-3b expression were found, compared to arterial tissue samples from healthy control individuals; furthermore, tRF-2 was only found to be expressed in tissue samples from controls. tRF-Gly-GCC expression is increased in atherosclerotic tissues and is involved in the pathogenesis of AS. This tRF regulates cell adhesion, proliferation, migration, and phenotypic transformation of endothelial cells and vascular smooth muscle cells. These findings provide an indication of the potential of tRFs as biomarkers and therapeutic targets for AS.

Coronary artery disease (CAD) is one of the most common causes of death worldwide. Coronary atherosclerosis causes luminal stenosis or occlusion, subsequently leading to myocardial ischemia, hypoxia, or necrosis, and ultimately to irreversible myocardial damage [74–85]. tRNA fragments are expressed at low levels in normal mouse heart tissues; however, expression is altered in RNA extracted from heart tissue at different times after death [6]. Further studies are needed to investigate the role of tsRNA in myocardial ischemia, but these molecules are also putative therapeutic targets for the treatment of this disease. Dhahbi et al. [86] identified 5′ tiRNA fragments in mouse serum resulting from tRNA degradation, whose expression levels changed with age and could be regulated by caloric restriction (CR). Liu et al. [87] found that 166 tsRNAs were upregulated and 136 tsRNAs were downregulated in heart tissue from a rat model of myocardial ischemia, indicating that the protective effect of CR against myocardial ischemia may be mediated by tsRNAs. In rat heart tissues undergoing CR, tRNA-His-GTG-004 was elevated and tRF-Cys-GCA-022, tRF-Lys-CTT-026, and tRF-Met-CAT-008 were reduced. These aberrantly expressed tsRNAs exert therapeutic effects by targeting genes such as Med13l, Sucla2, and Wls.

Myocardial hypertrophy is characterized by an increase in cardiomyocyte volume in the absence of cell division and is a major cause of morbidity and mortality in the elderly [88]. tsRNAs are involved in the regulation of myocardial hypertrophy through mechanisms including oxidative stress, aging, caloric intake, cellular ANG and ELAC2 expression, and hypoxia [89]. A recent study found that tRFs were highly expressed in a rat model of isoproterenol-induced cardiac hypertrophy, with the tRF-1 tRNA-Gly-CCC displaying the highest expression [90]. tRFs regulate myocardial hypertrophy by directly targeting the 3′ UTR of Timp3 via miRNA-like effects, and overexpression of both tRFs1 and tRFs2 expanded cardiomyocyte surface area and increased the expression of myocardial hypertrophy markers (atrial natriuretic factor, brain natriuretic peptide, and -myosin heavy chain).

### tsRNAs and bone and joint diseases

tsRNAs are involved in fracture healing. In a mouse model, expression of 5′ tiRNAs (Lys-CTT-5′ end, Lys-TTTT-5′ end) and i-tRFs (Lys-TTTT and Lys-CTT) was found to be reduced. Conversely, 5′ tiRNA (His-GTG-5′) and i-tRF (His-GTG) expression levels were increased during fracture healing and found to be involved in the regulation of osteogenesis and tissue remodeling through the Wnt/-catenin signaling pathway [91].

The incidence of osteoporosis (OP) and osteoarthritis (OA), major causes of workforce loss [92], has gradually increased with the aging population. A study of plasma exosomes from patients with OP found that exosome-derived tRF-25, tRF-38, and tRF-18 were expressed at higher levels in OP patients than in healthy controls, suggesting that these may be novel biomarkers for the diagnosis of OP [93]. Green et al. [94] found that tRF-3003a expression levels were increased in interleukin-1-stimulated OA chondrocytes. tRF-3003a can silence target gene expression and form a RISC by binding to the AGO2 protein in chondrocytes and targeting the 3′ UTR of JAK3 mRNA, thus acting like a miRNA. This suggests a potential regulatory role for this tRF in the pathogenesis of OA. Another study found that tRF-5009A could regulate autophagy and cartilage degeneration in OA by targeting the binding to the 3′ UTR of MTOR and inhibiting its expression [95]. These findings provide new directions for the study of cartilage degeneration and pathophysiological processes in OA.

### tsRNAs and autoimmune disease

Recently, studies have demonstrated altered tsRNA expression in the sera of patients with systemic lupus erythematosus (SLE) and lupus nephritis (LN) [96, 97], thus providing new clinical perspectives for the treatment of autoimmune diseases. Approximately 30% of patients with SLE develop LN, the clinical diagnosis of which relies on 24-h proteinuria and the presence of anti-double-stranded DNA (anti-dsDNA) antibodies. Yang et al. [98] found that tRF-His-GTG-1 was significantly expressed in sera from patients with SLE; tRF-His-GTG-1, in conjunction with anti-dsDNA, can be used as a biomarker for the diagnosis of SLE, with an area under the curve (AUC) of 0.95, a sensitivity of 83.72%, and a specificity of 94.19%. In addition, tRF-His-GTG-1 can be used as a non-invasive biomarker for the diagnosis of LN, with an AUC of 0.81, a sensitivity of 66.27%, and a specificity of 96.15%.

SLE pathogenesis is believed to be related to macrophage polarization. Recently, mesenchymal stem cells (MSCs) have shown great potential for the treatment of SLE [99, 100]. Dou et al. [101] found that tsRNA-21109 could be transferred through MSC-derived exosomes to inhibit the M1-type polarization of macrophages. The tsRNA-21109 target genes are enriched for macrophage activation-related signaling pathways, including Rap1, Ras, Hippo, Wnt, mitogen-activated protein kinase (MAPK), and transforming growth factor-, suggesting that tsRNA-21109 may be a novel target for the treatment of SLE.

IgA nephropathy (IgAN) is one of the most common forms of primary glomerulonephritis [102]. Numerous studies have found that interleukins, podocyte components, immune complexes, complement factors, and miRNAs in the peripheral blood or urine of patients with IgA are closely associated with the development of IgAN [103, 104]. Sequencing of peripheral blood mononuclear cells from patients with IgA and healthy controls showed differential expression of multiple tsRNAs, with upregulation of tRF-Val-AAC-007, tRF-Ala-AGC-063, tRF-Gln-CTG-010, tRF-Thr-AGT-007, and tRF-Tyr-GTA-011 and downregulation of tRNAs-Val-TAC-004, tRF-Gly-CCC-005, and tRF His-GTG-006 in patients [105]. These may be used as novel biomarkers for the diagnosis and treatment of IgAN.

### tsRNAs and metabolic disorders

The degradation fragment tRF-5c, of the tRNA carrying Gly, has been found to be the predominant type in porcine adipose tissue, which displays differential tsRNA expression in obese pigs versus lean pigs [106]. Furthermore, differentially expressed tsRNAs are involved in the regulation of energy metabolism, suggesting that, in pig adipocytes, tsRNAs may mediate the regulation of energy metabolism, promoting lipid deposition and leading to obesity. Wang et al. [107] first proposed that tsRNA-16902 is a novel regulator of adipogenesis that targets retinoic acid receptor to regulate lipogenic differentiation of hMSCs through the Smad2/3 signaling pathway. This pathway could thus be a potential novel target for the treatment of obesity. Another study, using a rat model of induced obesity [108] found that tRF-Glu-TTC inhibits adipogenesis, suggesting that tsRNAs of different lengths could mediate physiological processes with opposing effects.

### tsRNA and viral infection

A study has shown that 5′ tsRNA-Gly and 5′ tsRNA-Val, of approximately 30–35 nt in length, are significantly increased and exceed the abundance of miRNAs in liver tissues of humans and chimpanzees with chronic hepatitis B and C [109]. Furthermore, expression of tsRNA-20 (derived from tRNA-Gly-CCC-1–1) and tsRNA-46 (derived from tRNA-Glu-CTC-1–1) was downregulated [110]. Both of these tRNA fragments are produced from the 5′ end of tRNA. However, how tsRNAs are involved in HBV/HCV regulation remains unclear; further studies are needed to elucidate these mechanisms.

### tsRNA and lung injury

Acute lung injury (ALI) is caused by direct or indirect damage to alveolar epithelial cells and endothelial cells in capillaries, resulting in diffuse interstitial and alveolar edema. Despite advances in medical equipment and technology, half of the patients who develop ALI die [111]. Thus, there is an urgent need to identify novel molecular mechanisms and treatment strategies for ALI. A cytokine storm is the excessive and uncontrolled release of pro-inflammatory cytokines that can lead to severe inflammation and tissue damage and is a major contributor to the exacerbation of lung injury [112]. Exosomes from the bronchoalveolar lavage fluid of mice with ALI were primarily derived from alveolar macrophages (AMs) [113]. Activation of AMs is an important cause of ALI, and studies have found that tRF-22-8BWS7K092 derived from AM exosomes induces ferroptosis through activation of the Hippo signaling pathway, thus participating in the pathogenesis of this disease [113]. Understanding of the relationship between tsRNA and cytokine storm in lung injury is still emerging, and further research is needed to elucidate the mechanisms and clinical implications fully. The complex interplay between tsRNA, immune responses, and cytokine storm requires more investigation to determine the therapeutic potential of targeting tsRNAs for mitigating lung injury and cytokine storm. Moreover, Lin et al. [114] found that the expression of tsRNA-1020 and tsRNA-1018 was downregulated following dexmedetomidine treatment in patients with ALI and that this mechanism may be involved in the regulation of the nuclear-factor-B, MAPK, and phosphoinositide 3-kinase-Akt signaling pathways.

Radiation-induced lung injury (RILI) is one of the most common complications of thoracic radiotherapy and a major obstacle for improving the overall prognosis of patients with thoracic cancers [115]. The tRF-Gly-GCC has been found to mediate the development of RILI through inhibition of epithelial cell proliferation and promotion of reactive oxygen species production and apoptosis [116], suggesting that tsRNA may provide a novel perspective on the pathogenesis of RILI.

## Summary and outlook

This article summarizes current advances in research on tsRNAs in non-neoplastic diseases and provides insights into the functions and mechanisms of tsRNAs. However, research on this subject presents several problems. (1) A uniform standard nomenclature for tsRNAs must be established. (2) There exist differences in the direction of research for different types of tsRNAs. For example, there have been more studies on tRF than on tiRNA, which is evident from the fact that more tRF databases have been established, whereas there are fewer studies on i-tRF and tRF-2, relative to other types of tRFs, and their specific mechanisms remain unclear. (3) Unlike other non-coding small RNAs, tsRNAs inherit many modifications from the originating tRNA, which interfere with the subsequent detection of tsRNA expression. Therefore, these modifications should be removed from samples before sequencing, for the study of tsRNAs.

As an emergent topic in non-coding RNA research, tsRNA has potential but also faces challenges. It is believed that the gradually increasing attention on tsRNAs and improvements in sequencing technology will facilitate the discovery of more tsRNAs. Through in-depth investigation of the functions and regulatory mechanisms of tsRNA, their role in biology and medicine, as well as their role in the diagnosis and treatment of diseases, can be elucidated.

## Data Availability

All the data supporting the findings of this study are available from the corresponding author on reasonable request.
